# Morphological redescription and complete mitochondrial genome of *Deima oloughlini* (Holothuroidea, Synallactida, Deimatidae), with a new record from the northern Indian Ocean

**DOI:** 10.3897/zookeys.1286.190382

**Published:** 2026-07-29

**Authors:** Xiaomei Liao, Jun Li, Ningxia Xu, Xinlong Li, Meiling Ge, Tonghui Zhang, Min Li, Xuelei Zhang, Qinzeng Xu

**Affiliations:** 1 Key Laboratory of Marine Eco-Environmental Science and Technology, First Institute of Oceanography, MNR, Qingdao 266061, China Key Laboratory of Marine Eco-Environmental Science and Technology, First Institute of Oceanography, MNR Qingdao China; 2 School of Ocean Sciences, China University of Geosciences (Beijing), Beijing, 100083, China School of Ocean Sciences, China University of Geosciences (Beijing) Beijing China; 3 Laboratory for Marine Ecology and Environmental Science, Qingdao Marine Science and Technology Center, Qingdao 266100, China Laboratory for Marine Ecology and Environmental Science, Qingdao Marine Science and Technology Center Qingdao China

**Keywords:** 16S, COI, deep sea, *
Deima
*, holothurian, mitogenome, morphology, phylogeny, taxonomy

## Abstract

The family Deimatidae is widely distributed throughout the deep sea but remains poorly represented by both morphological and molecular data. During a 2022 scientific expedition to the Ninety East Ridge, Indian Ocean, two deep-sea holothurian specimens were collected at depths of 2447–2557 m. Following morphological re-examination together with phylogenetic analyses based on the mitochondrial COI and 16S genes, the specimens were identified as *Deima oloughlini* Mackenzie & Davey in Mackenzie et al. 2024, representing the first record of this species from the northern Indian Ocean. Detailed observations of ossicles from five body regions are provided to facilitate future taxonomic comparisons within the genus *Deima*. The complete mitochondrial genome of *D. oloughlini* was also characterized as a circular molecule of 16,263 bp containing 13 protein-coding genes, 22 transfer RNA genes, and two ribosomal RNA genes, with an overall A+T content of 72.2%. This mitogenome is among the few genomic resources currently available for Deimatidae and provides additional molecular data for future studies of the phylogeny, taxonomy, and biogeography of this deep-sea family.

## Introduction

The Class Holothuroidea (Phylum Echinodermata) is an ecologically significant and widely distributed group of marine benthic invertebrates, inhabiting environments from the intertidal zone to the hadal trenches (Purcell et al. 2016; Fernández-Rodríguez et al. 2019; Pierrat et al. 2022; Solís Marín et al. 2024). Despite their great ecological importance, holothurian taxonomy has historically been challenging, and the higher-level classification system has undergone several major revisions (Smirnov 2012; Miller et al. 2017). Traditional holothurian classification relied predominantly on external morphology (e.g., body shape, tentacle type, arrangement of tube feet and papillae) and internal anatomy, particularly the morphology of microscopic calcareous ossicles (Solís-Marín 2003; Pawson et al. 2010; Xiao and Zhang 2024). However, the widespread occurrence of morphological convergence (homoplasy) often confounds classification based solely on morphological characters (Miller et al. 2017). Notably, Miller et al. (2017) provided a comprehensive phylogenetic analysis of extant Holothuroidea based on multi-gene data, which revealed polyphyly in several traditional orders (e.g., Elasipodida and Aspidochirotida) and prompted a significant revision of their higher-level classification. In recent years, molecular phylogenetic studies utilizing mitochondrial (e.g., COI, 16S) and nuclear (e.g., H3, 18S, 28S) gene sequences have greatly advanced our understanding of evolutionary relationships within Holothuroidea (Wiklund et al. 2019; Yu et al. 2021; Pan et al. 2024). This integrative approach, combining morphological and molecular data, is increasingly used for species delimitation and classification in holothurians, particularly for deep-sea taxa (Xiao et al. 2023; Yuan et al. 2024).

The order Synallactida has three families: Stichopodidae Haeckel, 1896, Synallactidae Ludwig, 1894 and Deimatidae Théel, 1882. The phylogenetic relationships of Synallactida lack robust evidence, and further molecular data are required to elucidate them. Among complete mitochondrial genomes (mitogenomes) of the order Synallactida, the family Stichopodidae has been extensively investigated, whereas only a few mitogenomes from Synallactidae (Liao et al. 2020) and Deimatidae (Mu et al. 2025) have been published. Deimatidae was originally placed in Elasipodida (Hansen 1975), later transferred to the order Aspidochirotida (Smirnov 2012), and finally reassigned to Synallactida (Miller et al. 2017). While Hansen’s (1975) monograph remains a foundational resource of Deimatidae, the molecular data for this family are severely limited. Deimatidae is a small family that contains 17 accepted species within three genera (*Oneirophanta* Théel, 1879, *Orphnurgus* Théel, 1879, and *Deima* Théel, 1879). These deimatid holothurians are predominantly deep-sea dwellers, widely distributed from shallow waters to abyssal depths across the globe (Pawson 2002; Xiao and Zhang 2024). Records exist across all major oceans, including the Pacific, Atlantic, Indian, and Southern oceans (Solís Marín et al. 2024).

The genus *Deima* is a cosmopolitan inhabitant of bathyal and abyssal zones (Hansen 1975; Mackenzie et al. 2024), predominantly distributed in tropical to temperate regions (Walker et al. 1987; Rogacheva et al. 2013). In this study, two deep-sea holothurian specimens were collected by the submersible vehicle *Jiaolong* from the Ninety East Ridge in the northern Indian Ocean in 2022. Based on integrative taxonomic analyses that combined external morphology, ossicle characters, and mitochondrial COI and 16S rRNA gene sequences, the specimens were identified as *Deima oloughlini* Mackenzie & Davey, 2024, representing a new record of this species from the northern Indian Ocean. We further provide detailed morphological observations of external characters and ossicles from five body regions, together with the characterization of its complete mitochondrial genome. These data expand the known geographic distribution of *D. oloughlini* and contribute additional molecular resources to understand the taxonomy, phylogeny, and biogeography of Deimatidae in deep-sea ecosystems.

## Material and methods

### Specimen collection and morphological analyses

**Figure 1.**
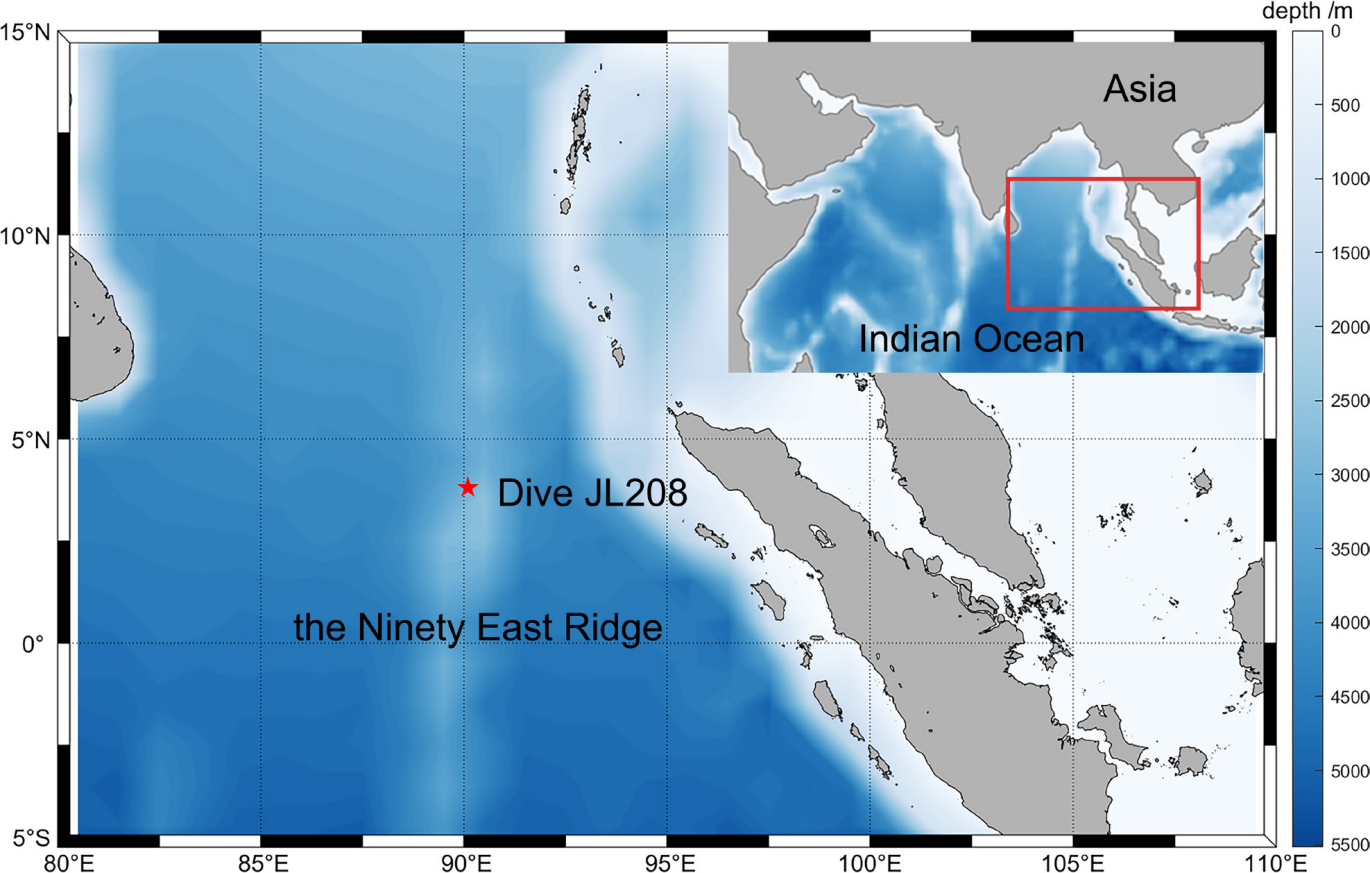
Sampling site for examined specimens of *Deima oloughlini* in the Ninety East Ridge, Indian Ocean.

Two deep-sea holothurian specimens were collected by *Jiaolong* human-operated vehicle in R/V *ShenHai YiHao* from the Ninety East Ridge in the Indian Ocean (Site 1: 3°48'59.07"N, 90°06'07.50"E, depth: 2557 m; Site 2: 3°48'13.20"N, 90°05'53.93"E, depth: 2447 m) on 9 May 2022 (Fig. 1). After the samples were collected from dive JL208, they were immediately photographed, dissected, rapidly frozen in liquid nitrogen, and stored at -80 °C for long-term preservation. The samples are deposited in the China Ocean Sample Repository at the First Institute of Oceanography, MNR, and their sample numbers are FIO-IND72-JLBEN208D1 and FIO-IND72-JLBEN208D2, respectively.

The specimens were identified based on comparisons of external and ossicle morphology with published descriptions, together with molecular phylogenetic evidence. External morphology was primarily examined macroscopically, aided by *in situ* images and photographs taken aboard the ship prior to dissection. Ossicle morphology was examined using both light microscopy and scanning electron microscopy (SEM). To extract ossicles, small pieces of tissue were taken from five body regions (dorsal body wall, ventral body wall, papillae, tube feet, and tentacles) of each specimen and digested in a ~10% sodium hypochlorite solution. After all the tissues were dissolved, the ossicles present in these tissues were rinsed three times with pure water and then three times with absolute ethanol. For SEM observation, ossicles were mounted on aluminum stubs using double-sided carbon tape to ensure proper conductivity. After air-drying, the stubs were sputter-coated with gold and observed using a Hitachi Regulus8100 SEM.

### mtDNA extraction and sequencing

Total genomic DNA was extracted from papillae using the E.Z.N.A. MicroElute Genomic DNA Kit (OMEGA BIO-TEK, USA) following the manufacturer’s protocol. Mitogenome sequencing was performed on an Illumina NovaSeq 6000 Platform (PE150, 350 bp DNA library) by Novogene Corporation (Beijing, China). The raw data were filtered by Trimmomatic v. 0.40 (Bolger et al. 2014) to obtain the clean reads for subsequent analysis.

The mitochondrial genome was assembled *de novo* using MitoZ v. 2.4-alpha (Meng et al. 2019). The initial assembly was verified by conducting BLAST searches against the NCBI nucleotide database to identify mitochondrial sequences for comparison. The circular mitogenome was annotated using MITOS2 (http://mitos2.bioinf.uni-leipzig.de/index.py) (Donath et al. 2019) for initial gene prediction and tRNAscan-SE 2.0 (http://lowelab.ucsc.edu/tRNAscan-SE/) (Lowe and Chan 2016) for tRNA gene identification. The alignment and correction of the entire mitogenome were conducted using MEGA v. 7.0 software (Kumar et al. 2016). To study the gene arrangement of mitochondrial genes, the phylogenetic analyses were performed using PhyloSuite v. 1.2.3 (Zhang et al. 2020; Xiang et al. 2023) and IQ-TREE v. 1.6.8 (Nguyen et al. 2015). The best-fit partitioning strategy and models were selected using ModelFinder (Kalyaanamoorthy et al. 2017) according to the Bayesian information criterion (BIC). ITOL v. 5.0 (Letunic and Bork 2021) was employed for the annotation and visualization of the phylogenetic tree.

### PCR amplification and Phylogenetic analyses

Following DNA extraction, the two mitochondrial cytochrome *c* oxidase I (COI) and 16S rRNA (16S) genes were amplified. The PCR amplification program for these genes is detailed in Table 1. All PCR amplification reactions were carried out in a 25 μL volume containing: 12.5 μL of 2× Taq PCR Master Mix (with blue dye, Sangon), 9.5 μL of ddH_2_O, 1.0 μL of each primer, and 1.0 μL template DNA. PCR products were visualized using 2% agarose gel electrophoresis and sequenced bidirectionally using Sanger sequencing at Sangon Biotech, Inc. (Qingdao, China), with the same primers. The resulting sequences were compared with the National Center for Biotechnology Information (NCBI) database.

**Table 1.** Primers and PCR conditions used in this Study. Cytochrome *c* oxidase subunit I (COI) was described by Arndt et al. (1996), 16S rRNA (16S) was described by Palumbi et al. (1991).

**Region**	**Primer**	**Sequence (5'-3')**	**PCR profile**
COI	COIef	ATAATGATAGGAGGRTTTGG	95 °C: 3 min, 40 cycles (95 °C: 40 s, 45 °C: 40 s, 72 °C: 50 s)
	COIer	GCTCGTGTRTCTACRTCCAT	
16S	16S-arL	CGCCGTTTATCAAAAACAT	94 °C: 4 min, 32 cycles (94 °C: 30 s, 56 °C: 30 s, 72 °C: 60 s)
	16S-brH	CCGGTCTGAACTCAGATCACG	

The first phylogenetic analysis was based on 24 partial COI sequences. This dataset included two sequences from this study (*D. oloughlini* JL208D1 and JL208D2) and 22 sequences downloaded from NCBI (21 from Deimatidae and one outgroup sequence from a species outside of Deimatidae but within Synallactida). To obtain robust phylogenetic inference, a separate analysis was conducted based on 16S rRNA gene sequences. This dataset comprised 25 partial 16S sequences (two from this study and 23 from NCBI). *Paelopatides* sp. (Synallactidae) was selected as the outgroup to root the phylogenetic tree. All sequences were aligned in batches with MAFFT v. 7.505 (Katoh and Standley 2013) and then trimmed with Gblocks v. 0.91 (Castresana 2000). The best-fit partitioning strategy and models were selected using ModelFinder (Kalyaanamoorthy et al. 2017). Maximum-likelihood (ML) phylogenies were inferred using IQ-TREE v. 2.2.0 (Nguyen et al. 2015) under the GTR+G+I model for 5000 ultrafast bootstraps. Pairwise genetic distances among Deimatidae species based on COI and 16S sequences were calculated in MEGA v. 7 using the Kimura 2-parameter (K2P) model, with 1000 bootstrap replicates used to assess statistical support.

## Results

### Taxonomy

**Order Synallactida Miller, Kerr, Paulay, Reich, Wilson, Carvajal & Rouse, 2017**

**Family Deimatidae Théel, 1882**

**Genus *Deima* Théel, 1879**

#### Deima oloughlini

Taxon classification

Animalia

SynallactidaDeimatidae

Mackenzie & Davey in Mackenzie et al. 2024

0DF9EC64-B140-5CC8-829D-691E199319E2

Figs 2, 3, 4

Deima oloughlini Mackenzie & Davey in Mackenzie et al. 2024: 264–266, fig. 37.

##### Material examined.

Two specimens. FIO-IND72-JLBEN208D1 (JL208D1), collected from Ninety East Ridge, Indian Ocean, JL208 (3°48'59.10"N, 90°06'07.50"E), depth 2557 m, 9 May 2022, preserved at -80 °C. FIO-IND72-JLBEN208D2 (JL208D2), collected from Ninety East Ridge, Indian Ocean, dive JL208 (3°48'13.20"N, 90°05'53.93"E), depth 2447 m, [9 May 2022], preserved at -80 °C.

**Figure 2.**
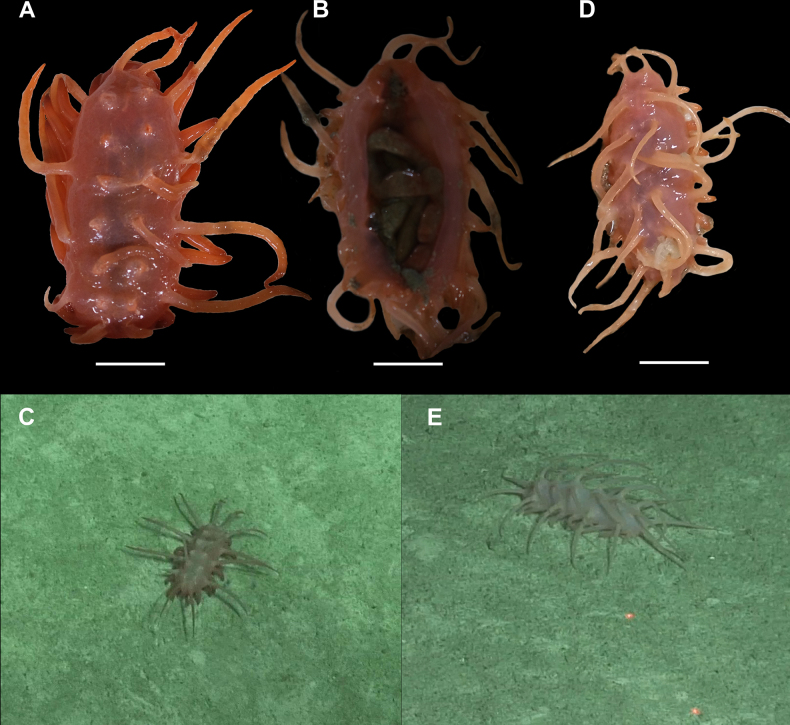
*Deima oloughlini*. **A–C**. FIO-IND72-JLBEN208D1 specimen: **A**. Dorsal view; **B**. Ventral view; **C**. *In situ* image; **D–E**. FIO-IND72-JLBEN208D2 specimen: **D**. Dorsal view; **E**. *In situ* image. Scale bars: 5 cm.

**Figure 3.**
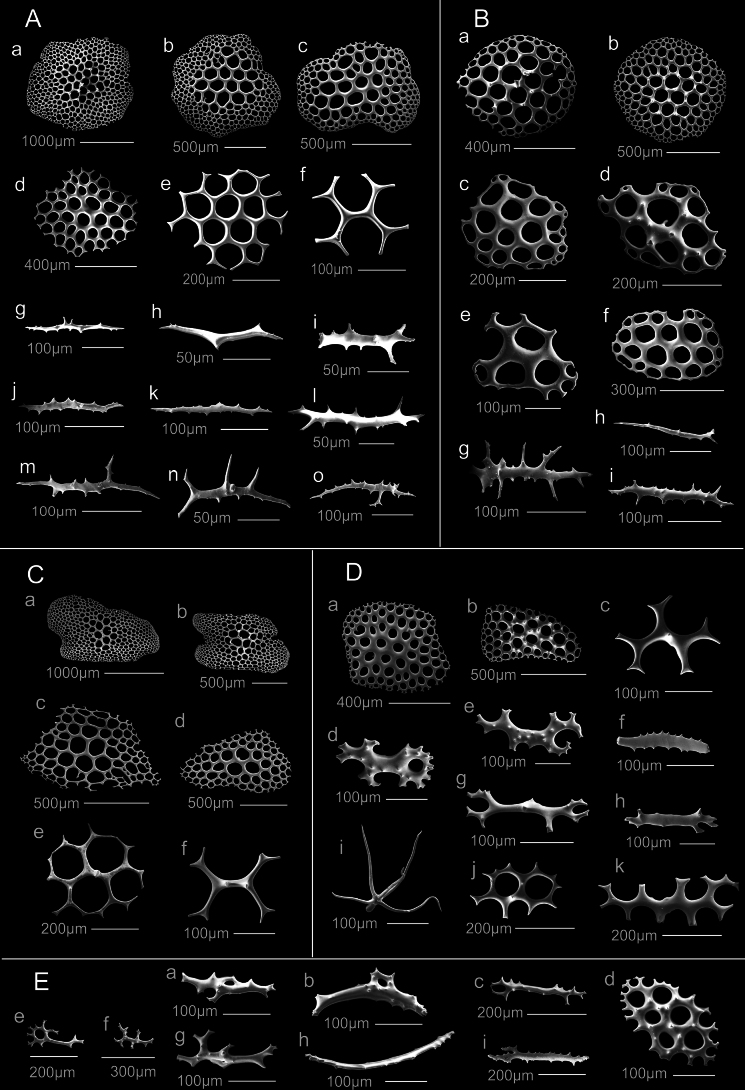
SEM images of different tissue ossicles from *Deima oloughlini* (FIO-IND72-JLBEN208D1). **A**. Dorsal body wall; **B**. Ventral body wall; **C**. Papillae; **D**. Tube feet; **E**. Tentacles.

**Figure 4.**
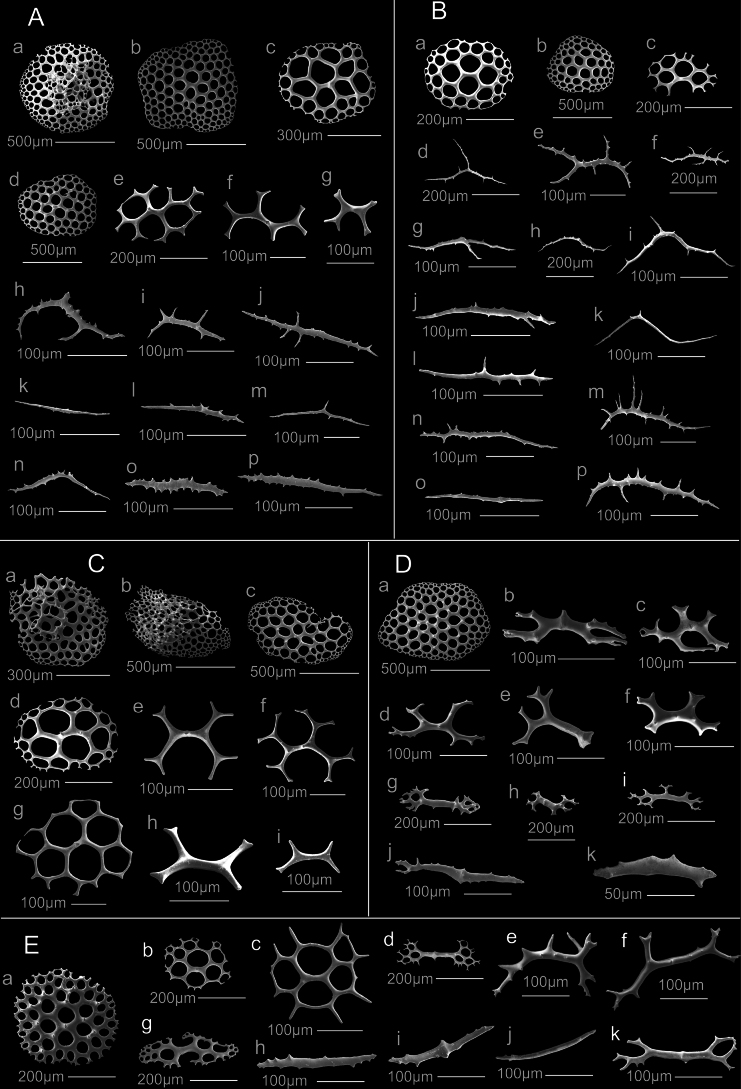
SEM images of different tissue ossicles from *Deima oloughlini* (FIO-IND72-JLBEN208D2). **A**. Dorsal body wall; **B**. Ventral body wall; **C**. Papillae; **D**. Tube feet; **E**. Tentacles.

##### Description.

***External morphology***. Body cylindrical, slightly flattened ventrally, skin smooth, color *in vivo* pink to reddish (Fig. 2C, E). The body width about 5–7.5 cm, and length approximately 13–17 cm (Fig. 2A, B, D). The base diameter of the dorsal papillae approximately 0.7–0.9 cm, with a length of 6–11 cm (Fig. 2A). Dorsal papillae flesh-pink, tips darker. Tube feet overall darker than papillae, tips red-black. Tube feet basal diameter approximately 700–1100 µm, length about 1600–3500 µm (Fig. 2A, B, D). Ventral mouth encircled by a raised, annular ridge, with retracted tentacles visible within. Dorsal papillae arranged in a bilateral symmetrical pattern. 6 pairs of slender medial papillae and 6 pairs of longer lateral papillae, all arranged symmetrically (Fig. 2A, B, D). Ventrolateral tube feet in approximately 11 on both sides of the abdomen. Tentacles retracted, number undetermined (Fig. 2A, B, D).

***Ossicle morphology***. Ossicles extracted from five body parts: dorsal body wall, ventral body wall, papillae, tube feet, and tentacles. The dorsal body wall deposits comprised three types of ossicles: perforated plates (Fig. 3A: a–e, diameter about 500–1900 µm); rods (Fig. 3A: g–o, 120–260 µm in length); and bifurcated crosses (Fig. 3A: f, 170–180 µm in length). The ventral body wall contained two types of ossicles (Fig. 3B: a–i). The perforated plates on the ventral side are smaller than those on the dorsal side, but some are also large, for example, one measuring ~1050 µm in diameter (Fig. 3B: b). Papillae contain perforated plates (Fig. 3C: a–d, diameter 1200–1800 µm), edges open or closed; bifurcated crosses (Fig. 3C: f, 200–280 µm in length) are also in papillae, similar to those in the dorsal body wall (Fig. 3A: f). Tube feet with several kinds of ossicles (Fig. 3D: a–k): perforated plates with central spikes (Fig. 3D: a, b, diameter 720–760 µm), bifurcated crosses (Fig. 3D: c, 210–220 µm in length), robust rods with spines (Fig. 3D: d, e, 230–300 µm in length), flattened rods with serrated edges (Fig. 3D: f, 190–200 µm in length, 15–35 µm in width), and rods bifurcated at both ends (Fig. 3D: g, h, 230–270 µm in length), along with three distinctively shaped sclerites (Fig. 3D: i–k). The tentacle deposits contained various rod-like structures (Fig. 3E: a–c, g–i, 230–430 µm in length) and two odd forms (Fig. 3E: e, f 220–240 µm in length), one of which was an irregular, branched rod-like ossicle (Fig. 3E: e). Smaller perforated plates with central spines (Fig. 3E: d, diameter 270–280 µm) are also present in the tentacles, appearing more compact compared to those found in other tissue sections.

##### Variability.

FIO-IND72-JLBEN208D2 (JL208D2) specimen body cylindrical; color *in vivo* pink. The JL208D2 specimen measured approximately 13 cm in length and 5 cm in width when collected, size smaller than JL208D1 (FIO-IND72-JLBEN208D1). Ossicles extracted from five body parts of the JL208D2 (Fig. 4). The dominant ossicles are perforated plates and various rods. However, some perforated plates with a distinctly three-dimensional architecture (Fig. 4A: a, Fig. 4C: a, b, diameter 540–840 µm), more reminiscent of table-shaped ossicles; several rods here more strongly curved and bearing markedly longer branches. Dorsal and ventral body wall with some very thin rods (Fig. 4A: l–n, Fig. 4B: h, k, o); tube feet and tentacles rods stout and ramified (Fig. 4D: b–f, k, Fig. 4E: e, f).

##### Distribution.

Indian Ocean, Australian IOT, Cocos (Keeling) Islands Territory, Cocos (Keeling) Stn and Rudist Seamount Stn, at depths of 1175–1896 m; the Ninety East Ridge, Indian Ocean, at depths of 2447–2557 m.

##### Remarks.

The examined specimens (FIO-IND72-JLBEN208D1 and FIO-IND72-JLBEN208D2) are assigned to *Deima oloughlini* Mackenzie & Davey, 2024 based on external morphology and ossicle characters. Both specimens conform to the diagnostic range of *D. oloughlini*, including 6 pairs of dorsal papillae and 4–6 pairs of ventrolateral papillae. Both JL208D1 and JL208D2 possess 6 pairs of dorsal papillae and 6 pairs of ventrolateral papillae. Tube feet are arranged in approximately 11 pairs, consistent with intraspecific variation reported for *D. oloughlini*. In the dorsal and ventral body wall, the ossicles are mainly irregular perforated plates, mostly single-layered and occasionally showing rudimentary secondary meshwork or branching; this condition agrees with the original description of *D. oloughlini* by Mackenzie et al. (2024).

Historically, the genus *Deima* has included six recognized species: *D. atlanticum* Hérouard, 1898, *D. blakei* Théel, 1886, *D. fastosum* Théel, 1879, *D. mosaicum* Ohshima, 1915, *D. validum* Théel, 1879; and *D. pacificum* Ludwig, 1894. The first five were treated as subspecies of *D. validum validum*, while *D. pacificum* was regarded as *D. validum pacificum*. In the present material, ossicles were examined separately from the dorsal body wall, ventral body wall, papillae, tube feet, and tentacles. In the dorsal and ventral body wall, the ossicles are mainly irregular perforated plates, mostly single-layered and occasionally showing rudimentary secondary meshwork or branching, which agrees with the original description of *D. oloughlini* by Mackenzie et al. (2024). Rod-like ossicles were also observed in several body regions, especially in the tube feet and tentacles. Because ossicle descriptions in previous *Deima* studies have often focused mainly on body-wall plates, these region-specific observations provide supplementary comparative information for *D. oloughlini* and other *Deima* taxa, but are not treated here as evidence for species-level separation.

Previous accounts of the genus have focused almost exclusively on perforated plates, with rods scarcely mentioned; in contrast, our specimens possess a striking variety of rod-shaped ossicles in the dorsal and ventral body walls, tube feet, and tentacles. Overall, the combination of region-specific ossicle composition and appendicular rod polymorphism is consistent with the assignment of the present material to *D. oloughlini*. The occurrence of this species at the Ninety East Ridge represents a new record from the northern Indian Ocean.

### Complete mitochondrial genome characterization of *Deima oloughlini*

**Figure 5.**
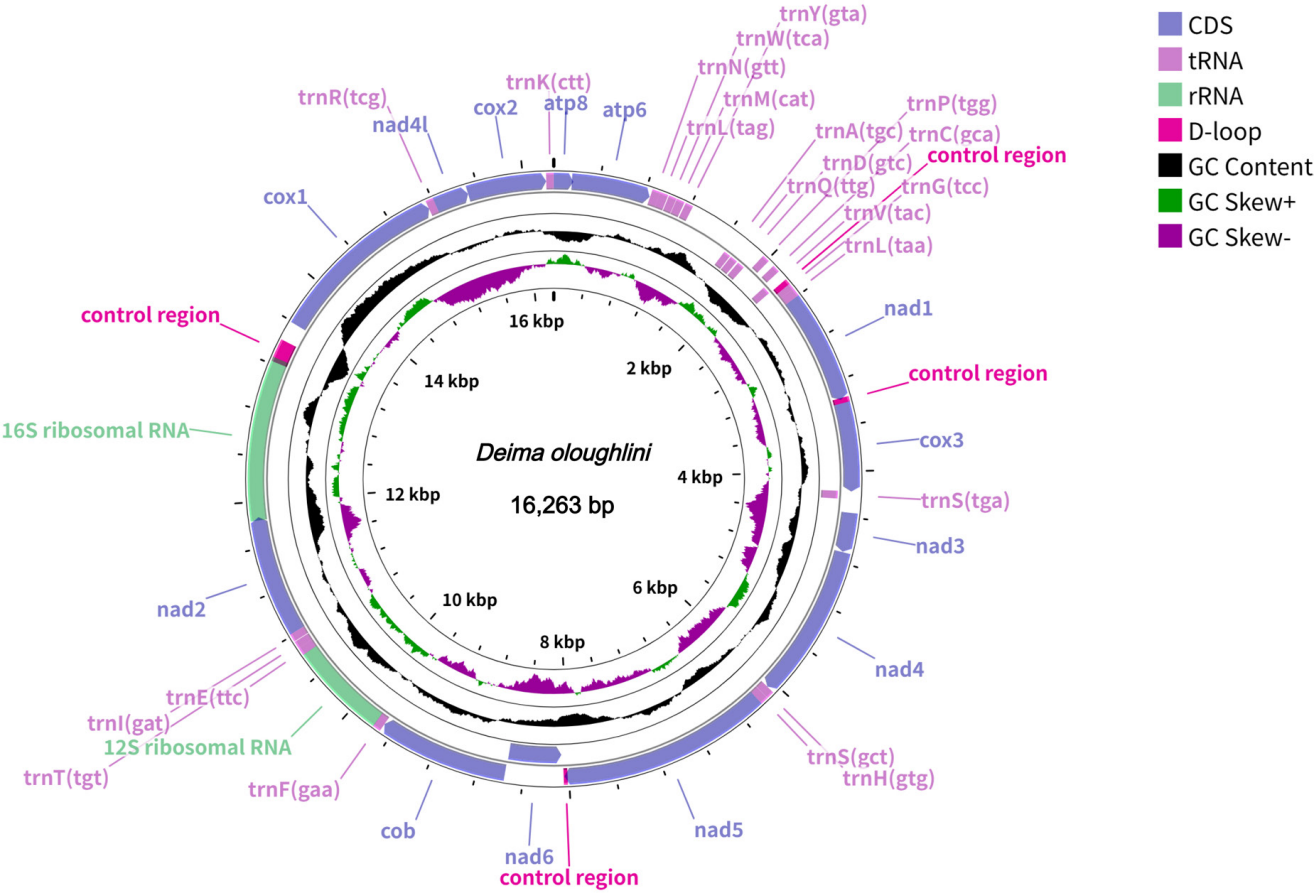
The complete mitochondrial genome of *Deima oloughlini*.

The mitochondrial genome size of *D. oloughlini* (GenBank accession: OQ503508) was 16,263 bp (Fig. 5). The mitogenome was circular, including 13 protein-coding genes (PCGs), 2 ribosomal RNA genes (rRNAs), and 22 transfer RNA genes (tRNAs). The overall base composition was 37.1% T, 35.1% A, 14.5% C, and 13.3% G. The overall A+T content was 72.2%, higher than that of most reported Synallactida species (Liao et al. 2020; Liu and Shen 2021). The mitogenome’s AT-skew (-0.0277) and GC-skew (-0.0431) both approached zero, reflecting an evolutionary equilibrium in nucleotide distribution between the complementary strands, suggesting a relatively stable architecture. All 13 PCGs shared a start codon ATG. Except for *cytb* and *nad4*, which use an incomplete stop codon (T--), all other PCGs terminate with a canonical TAA stop codon.

**Figure 6.**
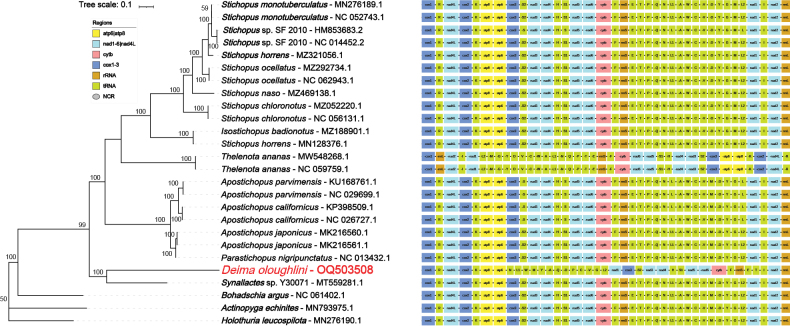
The phylogenetic relationships and mitochondrial genome arrangement.

The phylogenetic tree of Synallactida species was constructed using the concatenated sequences of 13 PCGs and 2 rRNAs. A total of 26 sea cucumber mitochondrial genomes were selected, including 22 from the same order as *D. oloughlini* (Synallactida) and three from order Holothuriida as the outgroup. The resulting phylogenetic tree recovered the order Synallactida as a monophyletic clade with high support, which is consistent with the current classification (Fig. 6). Within this clade, *D. oloughlini* formed a well-supported sister group to *Synallactes* sp. Y30071 (MT559281.1; Synallactidae). Comparative analysis revealed that the gene order of *D. oloughlini* differs from that of other species within the order Synallactida (Liao et al. 2020), indicating that gene rearrangements have occurred in this lineage.

### Molecular phylogeny and genetic distance based on the COI and 16S sequences

**Figure 7.**
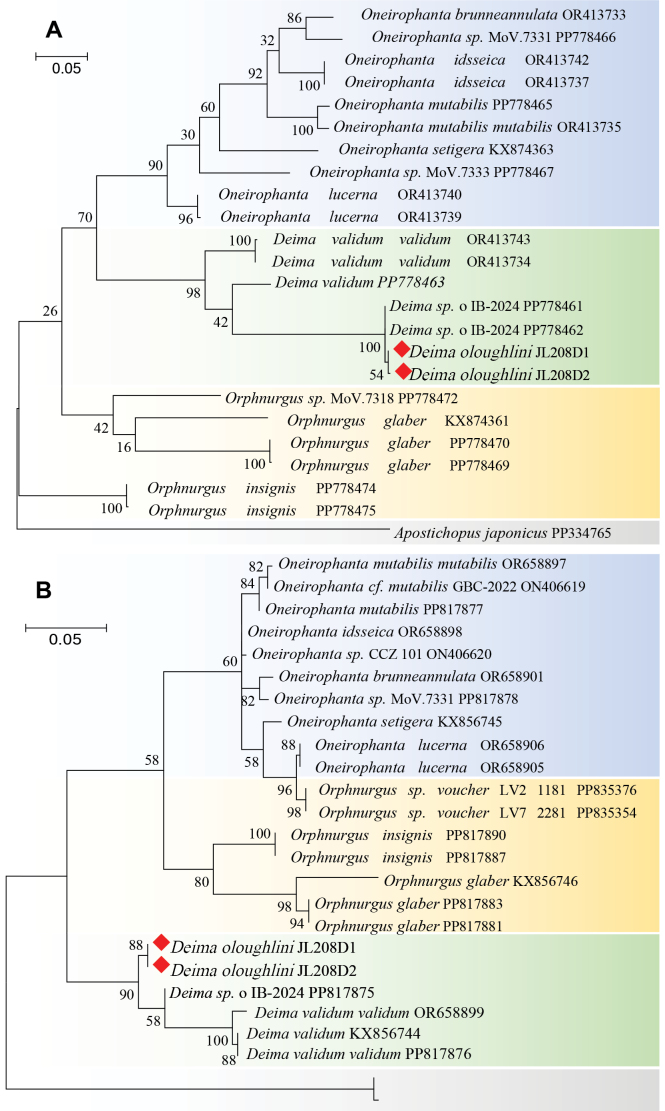
Maximum-likelihood (ML) trees based on COI (**A**) and 16S (**B**) sequences.

Maximum-likelihood (ML) phylogenies inferred from COI and 16S sequences were reconstructed to elucidate the intrageneric relationships within Deimatidae (Fig. 7). The COI tree (Fig. 7A) was constructed from 24 COI sequences, each 628 bp in length, and the 16S tree (Fig. 7B) employed 25 16S sequences, 425 p in length. The COI phylogeny robustly resolved the included deimatid holothurians into three well-supported clades (*Oneirophanta*, *Deima*, *Orphnurgus*; BS > 86), except for the outgroup – *Apostichopus japonicus* (Synallactida, Stichopodidae). The phylogenetic analysis revealed that specimens JL208D1 and JL208D2 formed a strongly supported clade (BS = 100) nested within the *Deima* lineage, closely associated with *Deima oloughlini* Mackenzie & Davey, 2024 (PP778461/PP778462). To confirm the phylogenetic placement based on COI, a separate analysis was performed using the 16S rRNA gene. The 16S tree robustly resolved three monophyletic genera of Deimatidae (*Oneirophanta*, *Deima*, *Orphnurgus*; BS ≥ 82), with *Paelopatides* (Synallactida, Synallactidae) as the outgroup. The 16S analysis also revealed that JL208D1 and JL208D2 form a clade with *Deima* sp. o IB-2024 (*D. oloughlini*) and are nested within the genus *Deima*.

Pairwise genetic distance analyses (Suppl. material 1) further supported the phylogenetic results. The intraspecific divergence between JL208D1 and JL208D2 was very low (K2P genetic distance: 0–0.24%), indicating close genetic affinity. The COI genetic distances between the present specimens and *Deima* sp. o IB-2024 sequences ranged from 0.5% to 0.7%, consistent with intraspecific variation reported for holothurians and supporting their clustering within *D. oloughlini*.

## Discussion

### Species identification and generic assignment

In this study, two specimens collected from the Ninety East Ridge in the Indian Ocean are identified as *Deima oloughlini* Mackenzie & Davey in Mackenzie et al. (2024). Following re-examination, the two specimens are consistent in the arrangement of papillae and tube feet, and they show ossicle characters consistent with the published description of *D. oloughlini*, dominated by perforated plates and rods occurring in comparable body regions. The low pairwise genetic distance between specimens JL208D1 and JL208D2, together with the low COI distance between the present material and *D. oloughlini* (0.5%–0.7%; Suppl. material 1), is more consistent with conspecificity than with species-level separation (~2% in typical interspecific comparisons).

Historically, the World Register of Marine Species (WoRMS 2026) officially recognized three extant species (including subspecies) within the genus *Deima*: *D. validum validum* (Théel 1879; Hansen 1975) (nominate subspecies), *D. validum pacificum* (Ludwig 1894) (a Pacific subspecies) and *D. oloughlini* (Mackenzie et al. 2024). However, the taxonomy of this genus has historically been complex. Prior to the revisions by Hansen (1975) approximately six species and subspecies were recognized, primarily distinguished by differences in dorsal papilla count and ossicle morphology. Hansen (1967) subsequently synonymized many of these into two subspecies: the near-cosmopolitan *D. validum validum* and the eastern Pacific *D. validum pacificum*. Our phylogenetic analyses firmly place both specimens within the genus *Deima*, showing strong support for their grouping with other *Deima* sequences (Fig. 7). The current evidence therefore supports identification as *D. oloughlini* rather than species-level separation. Morphological comparisons should be made using corresponding body regions, together with molecular evidence. Furthermore, previous descriptions of *Deima* have primarily emphasized perforated plates, whereas rod-shaped ossicles, especially those in appendicular regions such as tube feet and tentacles, have often been less completely documented. In the Ninety East Ridge specimens, rod-shaped ossicles occur in several body regions, especially in the tube feet and tentacles, together with perforated plates. These observations expand the available morphological information of *D. oloughlini*, especially for ossicles from appendicular regions such as tube feet and tentacles.

### Mitochondrial gene rearrangement and molecular phylogeny

The mitochondrial gene arrangement of the Ninety East Ridge material (Fig. 6), together with the rearranged gene order reported for *Thelenota ananas* (Stichopodidae) (Liu and Shen 2021), suggests that gene-order variation occurs within Synallactida and may be phylogenetically informative. The gene order observed here represents an additional arrangement among available synallactid mitogenomes. This complete circular mitogenome provides one of the few available genomic resources for Deimatidae and represents useful molecular data for difficult-to-access deep-sea taxa. It will facilitate future studies on molecular evolution, comparative mitogenomics, and phylogeography within Deimatidae and Synallactida. The mitochondrial genome reported here provides an additional data point for evaluating phylogenetic relationships within Synallactida. Mitochondrial gene arrangement may be phylogenetically informative, but it should be interpreted cautiously as a species-level taxonomic character given that genomic data for Deimatidae remain scarce. Thus, more mitogenomic data from additional species and genera are needed to understand the patterns and taxonomic utility of gene rearrangements within the group.

The phylogenetic trees (Fig. 7) placed JL208D1 and JL208D2 within *Deima*, where they were closely associated with *Deima* sp. o IB-2024 / *D. oloughlini* material from the Indian Ocean. The related Indian Ocean material reported by Mackenzie et al. (2024) was collected from the southeastern Indian Ocean, including the Australian Indian Ocean Territories around the Cocos (Keeling) Islands. The Ninety East Ridge specimens are consistent with this Indian Ocean material and extend the known distribution of *D. oloughlini* to the northern Indian Ocean. The region-specific ossicle observations provided here add comparative information for future studies of *D. oloughlini* and other *Deima* species. In addition, the placement of two *Orphnurgus* sp. sequences within the *Oneirophanta* clade in the COI tree suggests that some public sequences may require taxonomic verification.

## Conclusions

Based on an integrative taxonomic approach combining morphology and molecular phylogenetics, we identify two sea cucumber specimens from the Ninety East Ridge in the Indian Ocean as *D. oloughlini* (Deimatidae, Synallactida), representing a new record for the northern Indian Ocean. This identification is supported by the low COI genetic distance to *D. oloughlini* (0.005–0.007), phylogenetic placement within *Deima*, and corresponding external and ossicle characters. We provide a morphological redescription based on the Ninety East Ridge material, including detailed documentation of ossicles from five body regions, and report a complete mitochondrial genome for *D. oloughlini*. The mitochondrial gene order documented here adds comparative information for Synallactida and may be useful for future studies of mitogenomic evolution. The combined morphological and mitogenomic data provide a useful reference for future studies of deep-sea Deimatidae and Synallactida. These findings refine the known distribution and morphological variation of *D. oloughlini* and expand the genomic resources available for future evolutionary and phylogenetic studies of deep-sea holothurians.

## Supplementary Material

XML Treatment for Deima oloughlini

## Additional information

### Artificial Intelligence (AI) use

The authors accept full responsibility for the content of the manuscript, including the disclosure of any use of AI.

No AI tools were used in the preparation of this manuscript.

### Author ORCIDs

X. Liao https://orcid.org/0009-0004-7831-0524

J. Li https://orcid.org/0009-0002-6303-8068

N. Xu https://orcid.org/0009-0008-0975-5588

X. Li https://orcid.org/0009-0007-5924-9109

M. Ge https://orcid.org/0000-0001-7097-1783

T. Zhang https://orcid.org/0009-0003-2305-4052

M. Li https://orcid.org/0009-0001-4361-5828

X. Zhang https://orcid.org/0000-0002-3791-125X

Q. Xu https://orcid.org/0000-0003-3036-5361

### Data availability

All of the data that support the findings of this study are available in the main text or Supplementary Information.

## Supplementary materials

Supplementary material 1 Genetic distance of Deimatidae species based on COI and 16S genes, respectively Data type zip https://binary.pensoft.net/file/1730021This dataset is made available under the Open Database License (http://opendatacommons.org/licenses/odbl/1.0/). The Open Database License (ODbL) is a license agreement intended to allow users to freely share, modify, and use this Dataset while maintaining this same freedom for others, provided that the original source and author(s) are credited. Xiaomei Liao, Jun Li, Ningxia Xu, Xinlong Li, Meiling Ge, Tonghui Zhang, Min Li, Xuelei Zhang, Qinzeng Xu

Supplementary material 2 List of GenBank accession numbers used in this study Data type docx https://binary.pensoft.net/file/1730022This dataset is made available under the Open Database License (http://opendatacommons.org/licenses/odbl/1.0/). The Open Database License (ODbL) is a license agreement intended to allow users to freely share, modify, and use this Dataset while maintaining this same freedom for others, provided that the original source and author(s) are credited. Xiaomei Liao, Jun Li, Ningxia Xu, Xinlong Li, Meiling Ge, Tonghui Zhang, Min Li, Xuelei Zhang, Qinzeng Xu
