# Supplementary material for: Morphological redescription and complete mitochondrial genome of Deima oloughlini (Holothuroidea, Synallactida, Deimatidae), with a new record from the northern Indian Ocean
Source: Zookeys. 2026 Jul 29;1286:265–82. doi: 10.3897/zookeys.1286.190382 (PMC13444224; doi:10.3897/zookeys.1286.190382)
Supplement: Supplementary file 1 [file zookeys-1286-265_article-190382__-s002.docx]

| **Species** | **GenBank accession number** | |
| --- | --- | --- |
|  | **COI** | **16S** |
| **Deimatidae** | | |
| *Deima oloughlini* JL208D1 / JL208D2 | PZ085951 / PZ085952 | PZ096013 / PZ096013 |
| *Deima validum* | PP778463 | KX856744 |
| *Deima validum validum* | OR413734 / OR413743 | OR658899 / PP817876 |
| *Deima* sp. o IB-2024 | PP778461 / PP778462 | PP817875 |
| *Oneirophanta brunneannulata* | OR413733 | OR658901 |
| *Oneirophanta cf. mutabilis* | — | ON406619 |
| *Oneirophanta idsseica* | OR413737 / OR413742 | OR658898 |
| *Oneirophanta lucerna* | OR413739 / OR413740 | OR658905 / OR658906 |
| *Oneirophanta mutabilis* | PP778465 | PP817877 |
| *Oneirophanta mutabilis mutabilis* | OR413735 | OR658897 |
| *Oneirophanta setigera* | KX874363 | KX856745 |
| *Oneirophanta* sp. CCZ 101 | — | ON406620 |
| *Oneirophanta* sp. MoV.7331 | PP778466 | PP817878 |
| *Oneirophanta* sp. MoV.7333 | PP778467 | — |
| *Orphnurgus glaber* | KX874361 / PP778469 / PP778470 | KX856746 / PP817881 / PP817883 |
| *Orphnurgus insignis* | PP778474 / PP778475 | PP817887 / PP817890 |
| *Orphnurgus* sp. MoV.7318 | PP778472 | — |
| *Orphnurgus* sp. voucher LV2 1181 | — | PP835376 |
| *Orphnurgus* sp. voucher LV7 2281 | — | PP835354 |
| **Outgroups** | | |
| *Apostichopus californicus* | PP334765 | — |
| *Paeolopatides* sp. GMS-2020 | — | MW177886 |
| *Paeolopatides* sp. 1 AM-2017 | — | KX856757 |

**Suppl. material 2: 1. List of the GenBank accession numbers for all COI and 16S rRNA sequences used in this study.**

**2. List of the GenBank accession numbers for all mitochondrial genome used in this study.**

| **Family** | **Species** | **GenBank accession number** |
| --- | --- | --- |
| **Stichopodidae** | *Stichopus monotuberculatus* | MN276189.1 |
|  | *Stichopus monotuberculatus* | NC_052743.1 |
|  | *Stichopus* sp. SF 2010 | HM853683.2 |
|  | *Stichopus* sp. SF 2010 | NC_014452.2 |
|  | *Stichopus horrens* | MZ321056.1 |
|  | *Stichopus ocellatus* | MZ292734.1 |
|  | *Stichopus ocellatus* | NC_062943.1 |
|  | *Stichopus naso* | MZ469138.1 |
|  | *Stichopus chloronotus* | MZ052220.1 |
|  | *Stichopus chloronotus* | NC_056131.1 |
|  | *Isostichopus badionotus* | MZ188901.1 |
|  | *Stichopus horrens* | MN128376.1 |
|  | *Thelenota ananas* | MW548268.1 |
|  | *Thelenota ananas* | NC_059759.1 |
|  | *Apostichopus parvimensis* | KU168761.1 |
|  | *Apostichopus parvimensis* | NC_029699.1 |
|  | *Apostichopus californicus* | KP398509.1 |
|  | *Apostichopus californicus* | NC_026727.1 |
|  | *Apostichopus japonicus* | MK216560.1 |
|  | *Apostichopus japonicus* | MK216561.1 |
|  | *Parastichopus nigripunctatus* | NC_013432.1 |
| **Deimatidae** | *Deima oloughlini* | OQ503508 |
| **Synallactidae** | *Synallactes* sp. Y30071 | MT559281.1 |
| **Holothuriidae** | *Bohadschia argus* | NC_061402.1 |
|  | *Actinopyga echinites* | MN793975.1 |
|  | *Holothuria leucospilota* | MN276190.1 |
